# Scaling Up Psychological Treatments: A Countrywide Test of the Online Training of Therapists

**DOI:** 10.2196/jmir.7864

**Published:** 2017-06-16

**Authors:** Christopher G Fairburn, Elizabeth Allen, Suzanne Bailey-Straebler, Marianne E O'Connor, Zafra Cooper

**Affiliations:** ^1^ Department of Psychiatry University of Oxford Oxford United Kingdom; ^2^ London School of Hygiene and Tropical Medicine London United Kingdom; ^3^ Yale School of Medicine Department of Psychiatry New Haven, CT United States

**Keywords:** psychotherapy, training, dissemination, Internet, eating disorders, cognitive behavior therapy

## Abstract

**Background:**

A major barrier to the widespread dissemination of psychological treatments is the way that therapists are trained. The current method is not scalable.

**Objective:**

Our objective was to conduct a proof-of-concept study of Web-centered training, a scalable online method for training therapists.

**Methods:**

The Irish Health Service Executive identified mental health professionals across the country whom it wanted to be trained in a specific psychological treatment for eating disorders. These therapists were given access to a Web-centered training program in transdiagnostic cognitive behavior therapy for eating disorders. The training was accompanied by a scalable form of support consisting of brief encouraging telephone calls from a nonspecialist. The trainee therapists completed a validated measure of therapist competence before and after the training.

**Results:**

Of 102 therapists who embarked upon the training program, 86 (84.3%) completed it. There was a substantial increase in their competence scores following the training (mean difference 5.84, 95% Cl –6.62 to –5.05; *P*<.001) with 42.5% (34/80) scoring above a predetermined cut-point indicative of a good level of competence.

**Conclusions:**

Web-centered training proved feasible and acceptable and resulted in a marked increase in therapist competence scores. If these findings are replicated, Web-centered training would provide a means of simultaneously training large numbers of geographically dispersed trainees at low cost, thereby overcoming a major obstacle to the widespread dissemination of psychological treatments.

## Introduction

Psychological treatments are difficult to disseminate and implement [1,2]. They are inherently complex in form; they are labor-intensive and therefore costly; and, in contrast with drug treatments, there is no commercial drive to disseminate them. An additional obstacle is the way in which therapists are trained. This typically involves a therapist with generic training in psychological treatment delivery attending a specialist training workshop given by an expert and then practicing the treatment under the supervision of someone experienced at delivering the treatment [3]. As there is a scarcity of people qualified to provide both the workshops and the clinical supervision, this method is incapable of meeting the worldwide demand for training [4].

To address these problems, Web-centered training was devised [4]. Two main features distinguish it from conventional training. First, the training is program-led; that is, the training program itself leads the therapist through the training obviating the need for input from an external expert. Second, by delivering the training over the Internet, large numbers of geographically dispersed therapists can be trained at one time.

This investigation was a proof-of-concept study with 2 aims. The first was to determine whether Web-centered training is feasible and acceptable to therapists. The second aim was to establish its likely effectiveness. In common with most large-scale interventions, Web-centered training was not expected to be as potent as more intensive methods [5], a shortcoming that would be offset by its far greater scalability.

## Methods

### Design and Context

This was a proof-of-concept study in which a cohort of therapists across Ireland was offered Web-centered training in a specific psychological treatment. Their competence was assessed before and after the training.

The opportunity to undertake the study arose as a result of the Irish Health Service Executive (HSE) deciding to establish a countrywide eating disorder service. This necessitated identifying mental health professionals from across the Republic of Ireland whom the HSE wished to receive training in enhanced cognitive behavior therapy for eating disorders (CBT-E) [6,7]. CBT-E was chosen by the HSE as its treatment of choice because it has a strong evidence base across the full range of eating disorders, a decision consonant with the 2017 National Institute for Health and Care Excellence guidelines on eating disorders [8]. The Centre for Research on Eating Disorders at Oxford (CREDO) was asked to provide the training because it developed CBT-E [6]. Fortuitously, this request came at a time when CREDO was completing the development of a Web-centered training program in CBT-E. It therefore provided an opportunity to conduct a proof-of-concept study of the new method of training.

The research protocol was submitted to Oxford University Central Research Ethics Committee. It was judged not to require formal ethical approval.

### Recruitment of the Trainees

**Figure 1:**
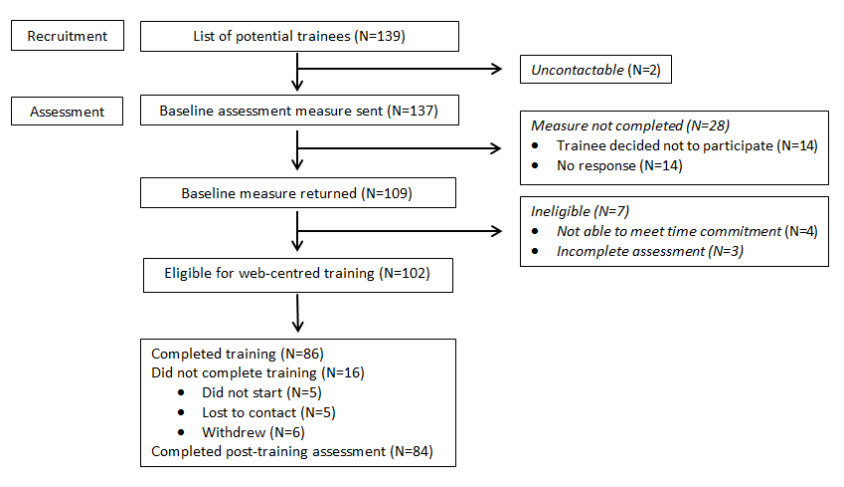
Recruitment and retention of the trainees.

The HSE identified 139 therapists as potential candidates for training. There were no formal inclusion or exclusion criteria. Each of these therapists was sent an email from CREDO in which they were invited to take part in an evaluation of the new form of training. They were provided with details about CBT-E and the study (Multimedia Appendix 1) together with an outline of the content of the training website (Multimedia Appendix 2). Those therapists who indicated that they were willing to take part and who provided informed consent were asked for details of their professional background, age, gender, and clinical experience. They were then given access to the training program for 20 weeks. Figure 1 shows the figures for the recruitment and retention of the trainees.

### The Web-Centered Training Program

The Web-centered training program in CBT-E has 2 main parts, The Course and The Library. The Course is linear in nature and takes a minimum of 9 hours to complete (Multimedia Appendix 2). It provides a detailed practical description of how to implement the main focused form of CBT-E given by an expert on the treatment (CGF). This description takes the form of multiple brief video presentations accompanied by handouts and separated by formative learning exercises, video recordings of acted illustrations of the treatment, and tests of knowledge accompanied by feedback. While working through The Course, trainees are encouraged to read relevant sections from the CBT-E treatment guide [6] and treat 1 or 2 patients. As implementing CBT-E with patients who are not severely underweight takes up to 20 weeks, access to the program was for this length of time. It was stressed to the trainees that clinical responsibility for their patients remained with their local clinical team and not with CREDO.

The second part of the training website, The Library, provides supplementary information on how to use CBT-E with subgroups of patients including those who are severely underweight and those with clinical perfectionism, core low self-esteem, or marked interpersonal difficulties. There is also a detailed account of how to use CBT-E to treat young patients. At the time of conducting this study, The Library was not available but trainees had full access to all the material of relevance to the implementation of the focused version of CBT-E.

Web-centered training is designed to be undertaken ether autonomously (independent training) or with support from a nonspecialist support worker (supported training). Although the supported form requires external input, it is still scalable because the role of the support worker is restricted to encouraging the trainee to follow the training program. In this study the supported form of training was used. Thus, in addition to having access to the website, the trainees were offered up to 12 telephone calls over the 20 weeks of training, each lasting no longer than 30 minutes. These calls were weekly at first and then every 2 weeks. The support workers followed a protocol that defined their role, and they were supervised by 2 senior clinicians (ZC and SBS); 2 of the support workers were research assistants with no clinical experience and 2 had clinical experience but restricted themselves to the supportive role.

### Assessment of Competence

The competence measure was developed in advance of the study. It was designed to be scalable in nature so that it could be used in large-scale training studies such as this one. Its development and validation are described in detail in a separate publication [9]. This included detailed blueprinting, state-of-the-art item writing, and independent item review with initial field testing, followed by formal Rasch analysis. The measure was developed independently of the Web-centered training program in order to avoid teaching to the test.

The final version of the measure comprises 22 multiple choice questions, each of which addresses the trainee’s knowledge and understanding of the focused version of CBT-E and its implementation (ie, applied knowledge). Total scores on the measure have been related to performance at implementing CBT-E, and there is a cut-point indicative of a good level of competence at delivering CBT-E. Three equivalent versions of the measure are available so that different versions can be used before and after training.

### Data Analysis

Regression analysis was used to estimate the mean difference in therapist competence scores and to explore whether trainee characteristics (age, gender, professional background, years of clinical experience), support characteristics (support worker background, number of supportive phone calls), and the trainee’s use of a practice case were associated with change. Logistic regression (adjusted for baseline score) was used to explore factors associated with the likelihood of trainees scoring over the competence cut-point following the training.

## Results

### Participants

Of the 139 potential participants identified by the HSE, 102 embarked upon Web-centered training (Figure 1), 93 of whom were female. Their mean age was 40.1 (SD 8.1) years, and their mean length of clinical experience was 12.0 (SD 7.9) years. The professional background of the trainees is shown in Table 1. They comprised 2 main professional groups, clinical psychologists (37.3%) and psychiatric nurses or nurse therapists (34.4%). Three-quarters (74.4%) currently treated patients with eating disorders, although a third (33.3%) did so rarely.

**Table 1:** Professional background of the trainees (N=102).

Profession	Number n (%)
Clinical psychologist	38 (37.3)
Dietitian	3 (2.9)
Family therapist	1 (1.0)
Nurse therapist	2 (2.0)
Occupational therapist	9 (8.8)
Psychiatric nurse	33 (32.4)
Psychiatrist	3 (2.9)
Social worker	13 (12.7)

### Completion of Training

Of 102 trainees, 86 (84.3%) completed the full training program. Of the remainder, 11 failed to start (6 cited practical reasons for withdrawing), and 5 were lost to contact (Figure 1).

### Outcome of Training

Of the 86 trainees who finished the entire training program, 80 completed the competence measure before and after their training. Their scores increased substantially from 5.39 before training (95% CI 4.78-6.00) to 11.23 after training (95% CI 10.46-11.99), the mean difference being 5.84 (95% Cl –6.62 to –5.05, *P*<.001). One of these trainees scored above the competence cut-point prior to the training (1.3%, 1/80) whereas 34 did so afterwards (42.5%, 34/80).

The exploratory logistic regression analyses identified 1 significant predictor of outcome. Psychiatric nurses and nurse therapists had a smaller increase in their competence scores than the other professional groups (mean difference 2.52, *P*=.003) and were less likely to score above the cut-point (OR 0.17, 95% CI 0.05-0.58; *P*=.005).

## Discussion

### The Study and its Findings

This study was an opportunistic initial evaluation of a new scalable way of training therapists to deliver specialist psychological treatments. It capitalized on a training initiative of the Irish HSE. It found that Web-centered training, when accompanied by nonspecialist support, was practicable, acceptable, and reasonably effective. Over 80% of the trainees completed the training program, and their scores on the validated measure of competence increased substantially. Almost half obtained competence scores indicative of a good level of competence.

Certain practical difficulties were encountered. One was technical. A beta version of the website was used and this proved unstable at times. The other was logistic. Some trainees reported difficulties implementing the training during their working hours. This was for 2 main reasons. First, given their clinical commitments, it was difficult to protect time for the training and second, in some rural areas there was limited access to the Internet. Both difficulties proved surmountable.

### Comparison with Other Training Studies

It is difficult to provide a context within which to interpret these findings because therapist training has been relatively neglected as a research topic [10,11]. Furthermore, many of the training studies to date have been small in scale and few have used competence measures with a validated cut-point [12]. The competence figures that have been reported following training vary widely with higher figures coming from training programs that have included extensive supervision, a method that is not scalable and therefore of little relevance in this context.

### Strengths of the Study

The study had several strengths. First, it was conducted in a real-world setting and should therefore be viewed as an effectiveness study rather than an efficacy one. Second, the sample size was large for a training study although it was too small to draw firm conclusions regarding subgroups within the sample. This qualification particularly applies to the exploratory predictor analyses. Third, it used a validated measure of therapist competence.

### Limitations of the Study

The study had certain limitations. First, the trainees were selected by the HSE rather than being volunteers as would usually be the case. Second, there was no posttraining follow-up to determine whether the effects of the training persisted and whether the trainees put into practice what they had learned. Third, the study was not controlled. We cannot therefore discount the possibility that the increase in therapist competence scores would have happened spontaneously although this seems improbable. Nor do we know how this form of training would compare in effectiveness with other training methods. A comparison with the conventional method of training might seem the obvious design but we believe that the findings would be of limited interest given the poor scalability of the latter method. Instead, a comparison of independent and supported training would be of greater value as both are scalable yet one has higher inherent costs and is harder to implement.

### Conclusions

Two findings emerge from this opportunistic study. First, Web-centered training is feasible and well accepted. Over 80 therapists, dispersed across a wide geographical area, completed the training program despite receiving only a limited amount of nonspecialist support. Training on this scale, and at a distance, would be impossible with the current method of training. Second, the training was reasonably effective as almost half the trainees obtained a competence score indicative of a good level of competence. While this figure is lower than that reported with more intensive but unscalable forms of training, it is impressive given the scalability of the new method.

If these findings were replicated, Web-centered training would provide a means of simultaneously training large numbers of geographically dispersed therapists thereby overcoming a major obstacle to the dissemination of psychological treatments.

## Appendix

Multimedia Appendix 1 Overview of enhanced cognitive behavior therapy for eating disorders.

Multimedia Appendix 2 The training program.

Multimedia Appendix 3 Web-centred training.

## Abbreviations

CBT-E: Enhanced cognitive behaviour therapy for eating disorders
CREDO: Centre for Research on Eating Disorders at Oxford
HSE: Health Service Executive
NICE: National Institute for Health and Care Excellence
