# Supplementary material for: Scaling Up Psychological Treatments: A Countrywide Test of the Online Training of Therapists
Source: J Med Internet Res. 2017 Jun 16;19(6):e214. doi: 10.2196/jmir.7864 (PMC5493785; doi:10.2196/jmir.7864)
Supplement: Multimedia Appendix 1 [file jmir_v19i6e214_app1.pdf]

# OVERVIEW OF CBT-E

## INTRODUCTION

CBT-E is a psychological treatment designed for people with eating disorder psychopathology. It is primarily an outpatient treatment, but it has been adapted for inpatient and day patient settings. It can be used with adults and younger patients. The treatment is described in detail in the treatment guide (Fairburn, 2008).

When learning CBT-E it is best to concentrate on mastering the core form of the treatment (the 20-session, "focused" form). [See Appendix below for further explanation.] Once this form of the treatment has been mastered you can move onto its lengthier version (designed for underweight patients), its "broad" form, and how it is adapted to suit adolescents.

## THE TREATMENT

There are four stages in CBT-E. These are shown diagrammatically in the "CBT-E Map" overleaf. These stages differ in their duration and the number of sessions devoted to them (see table below).

The treatment starts intensively with a four-week block of twice-weekly sessions. Then there is a run of 10 weekly sessions. These are followed by three concluding sessions which are held every two weeks. Finally, there is a post-treatment review session 20 weeks after completing treatment.

| | **Week in Treatment** | **Number of Sessions** |
|---|---|---|
| **Stage One** | 1, 2, 3, 4 | Initial session, plus seven twice-weekly sessions |
| **Stage Two** | 5 | Usually one weekly session |
| **Stage Three** | 6, 7, 8, 9, 10, 11, 12, 13, 14 | Nine weekly sessions |
| **Stage Four** | 16, 18, 20 | Three bi-weekly sessions |

**Stage One (Starting Well)**

This is the initial stage of treatment. It is important that it is done well as it forms the foundation for the rest of treatment. The sessions in Stage One are twice-weekly over four weeks.

Stage One has many components. It has four primary aims:

1. To engage patients in CBT-E and the need for change, and to maintain their engagement thereafter
2. To increase their understanding of their eating problem
    i. By creating a personalised formulation concerning the processes that are likely to be maintaining their eating problem
    ii. By engaging in the real-time self-monitoring (tracking) of relevant psychopathology
    iii. By providing personalised education of relevance to the patient's eating problem
3. To educate the patient about body weight and weight change, and to reduce concerns about weight
4. To introduce and establish a stable pattern of regular eating

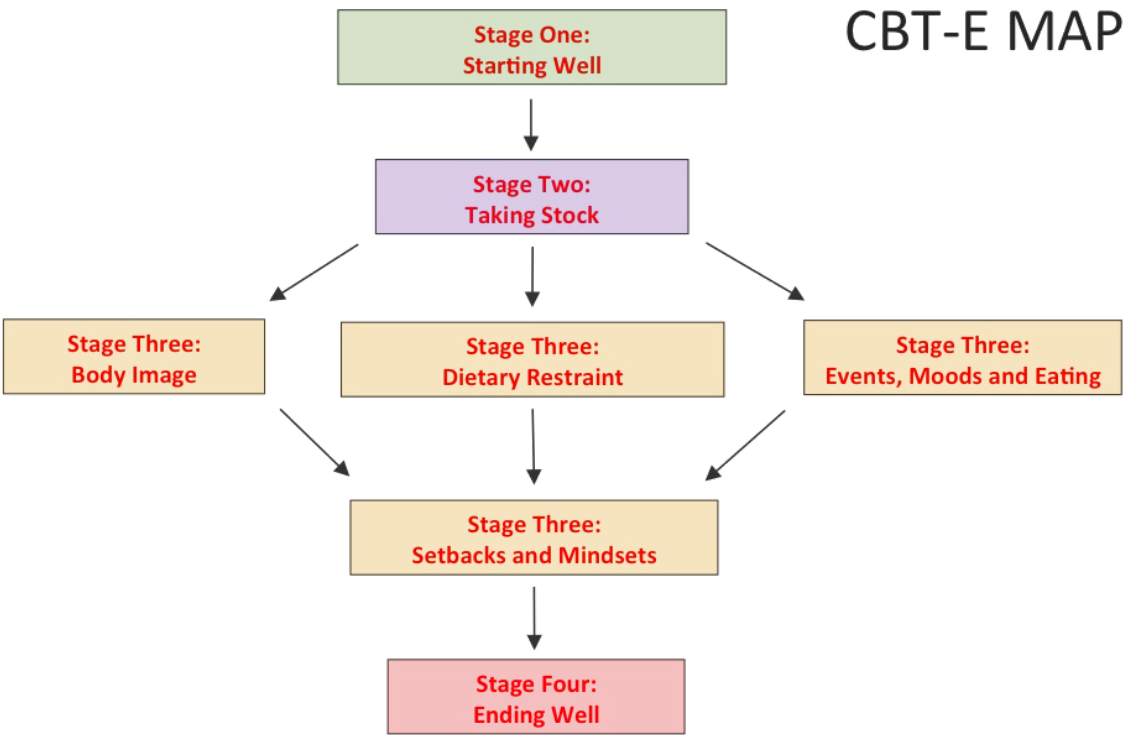


**Stage Two (Taking Stock)**

This is a brief transitional stage. It usually occupies one session and it is at this point that the sessions start to be weekly. The strategies and procedures introduced in Stage One continue.

The aims of Stage Two are as follows:

1. To review progress in Stage One (in terms of the patient's engagement, compliance and treatment response)
2. To review the formulation and modify it if need be.
3. To decide whether there is a need to use the broad form of the treatment
4. To plan Stage Three
   i. To decide which of the three core Stage Three treatment modules will need to be used (see the CBT-E map)
   ii. To decide the order in which they will be implemented

**Stage Three (Addressing the Maintaining Mechanisms)**

Stage Three takes place over a run of about 10 weekly sessions. It is highly personalized and involves the planned introduction and use of one or more of the following three treatment modules (see CBT-E map):

- Body image
- Dietary restraint
- Events, moods and eating

These modules are designed to address the processes maintaining the patient's eating problem.

Towards the end of Stage Three the treatment becomes more future-focused and starts to address how to deal with setbacks. Setbacks are common when emerging from an eating problem and they can lead to full-scale relapses. It is therefore important to help patients spot setbacks early and address them promptly.

**Stage Four**

Stage Four comprises three sessions held at two-week intervals. It has three aims:

1. To phase out certain treatment procedures (self-monitoring; in-session weighing)
2. To devise a plan for maximising the likelihood that the changes obtained will the maintained following the end of treatment
3. To devise a plan for dealing with setbacks in the future (should they occur).

**The Post-Treatment Review Session**

This is a single session held 20 weeks after the completion of treatment. Its aims are as follows

1. To review the patient's progress
2. To address any problems that remain or have reoccurred

In most cases this session marks the end of CBT-E.

**Appendix**

There are two versions of CBT-E:

- 20-session version - This version is suitable for the great majority of patients. It is designed for patients who do not need to regain significant weight as part of their treatment (i.e., those with a BMI ≥18.0).
- Extended version - This version is for patients who do need to regain weight (BMI 15.0 - <18.0) as part of their treatment. Its exact duration depends upon the amount of weight that needs to be regained. It is often in the region of 40 weeks.

There are also two forms of CBT-E:

- The focused form - This concentrates almost exclusively on the modification of the eating disorder psychopathology (the disturbed eating habits and the extreme concerns about shape and weight).
- The broad form - This is a more complex form of the treatment designed for a subgroup of patients (about 25% in most settings). In addition to addressing the eating disorder psychopathology, this form of CBT-E also addresses one or more of the following obstacles to change - clinical perfectionism, core low self-esteem and marked interpersonal problems.

The decision about whether to use the 20-session version or the extended one can be made at the outset of treatment. In contrast, the decision whether to use the focused or broad form is delayed until Stage Two of CBT-E.

**The Treatment Guide**

Fairburn CG. Cognitive behaviour therapy and eating disorders. Guilford Press, New York, 2008.
