# Supplementary material for: Scaling Up Psychological Treatments: A Countrywide Test of the Online Training of Therapists
Source: J Med Internet Res. 2017 Jun 16;19(6):e214. doi: 10.2196/jmir.7864 (PMC5493785; doi:10.2196/jmir.7864)
Supplement: Multimedia Appendix 2 [file jmir_v19i6e214_app2.pdf]

THE CBT-E TRAINING PROGRAMME

This web-based CBT-E training programme is designed to help you implement CBT-E well. It centres on a large collection of video presentations given by an expert on the treatment, and it is in three sections, **The Introduction**, **The Course** and **The Library**. The Introduction is a brief account of the nature and style of CBT-E. [CBT-E is described in outline in the accompanying handout "*Overview of CBT-E*."] The Course is a detailed and highly practical description of how to implement CBT-E. As its name implies, it is linear in its organisation and additive in nature. It is therefore important that you work through The Course in the order in which the material is presented. In contrast, and as its name implies, The Library is a repository of training material that can be accessed at any time.

The Introduction

The Introduction is a brief overview of CBT-E lasting about 40 minutes. It addresses the following topics:

- The indications for CBT-E and its goals
- The formulation (case conceptualisation)
- The duration of CBT-E and its two forms
- The style of CBT-E
- Preparing patients for CBT-E and implementing it well
- The structure and content of CBT-E

The Course

The goal of The Course is to help you implement the focused form of CBT-E (20-session version). The four stages of CBT-E, and their component modules, are described in turn with each one being the focus of one or more tutorials. Most of the tutorials last between 20 and 30 minutes and each comprises a video-based description of the topic concerned accompanied by clinical illustrations, learning exercises and handouts in the form of downloadable PDFs. The total length of The Course is about nine hours. The table overleaf lists the tutorials in The Course together with their duration.

The Library

The Library is an resource to be dipped into at will. It contains the following material:

1. All the material in The Course
2. Additional modules, including ones on the following topics:
    - Younger Patients
    - Underweight patients (anorexia nervosa)
    - Mood intolerance
    - Broad form of CBT-E
        - Clinical perfectionism
        - Core low pelf-esteem
        - Marked interpersonal problems

3. A large number of additional clinical demonstrations. These are longer than those embedded within The Course.

The Library is indexed to facilitate searching.

**WEB-CENTRED TRAINING IN CBT-E**<br>**Content and Timings**	
**THE INTRODUCTION (about 1 hour)**	
Overview of the training website<br>Overview of the treatment	10 mins<br>40 mins
**THE COURSE (about 9 hours in total)**<br>**Stage One (2 hrs 40 mins)**	
<u>Starting Well Module</u><br>The initial session<br>Creating a formulation<br>Establishing self-monitoring<br>Weight, weighing and weight concern<br>Establishing regular eating	 40 mins<br>20 mins<br>30 mins<br>40 mins<br>30 mins
**Stage Two (40 mins)**	
<u>Taking Stock Module</u><br>Reviewing progress<br>Planning Stage Three	 20 mins<br>20 mins
**Stage Three (4 hrs 20 mins)**	
<u>Body Image Module</u><br>Over-evaluation of shape and weight<br>Developing other domains<br>Body checking<br>Comparison-making<br>Body avoidance<br>Feeling fat<br><u>Events, Moods and Eating Module</u><br><u>Dietary Restraint Module</u><br><u>Setbacks and Mindsets Module</u>	 40 mins<br>15 mins<br>35 mins<br>20 mins<br>20 mins<br>25 mins<br>25 mins<br>50 mins<br>30 mins
**Stage Four and the Post-Treatment Review Session (1 hour)**	
<u>Ending Well Module</u>	30 mins

Post-treatment Review Session	30 mins
