# Supplementary figures and images for: Scaling Up Psychological Treatments: A Countrywide Test of the Online Training of Therapists
Source: J Med Internet Res. 2017 Jun 16;19(6):e214. doi: 10.2196/jmir.7864 (PMC5493785; doi:10.2196/jmir.7864)

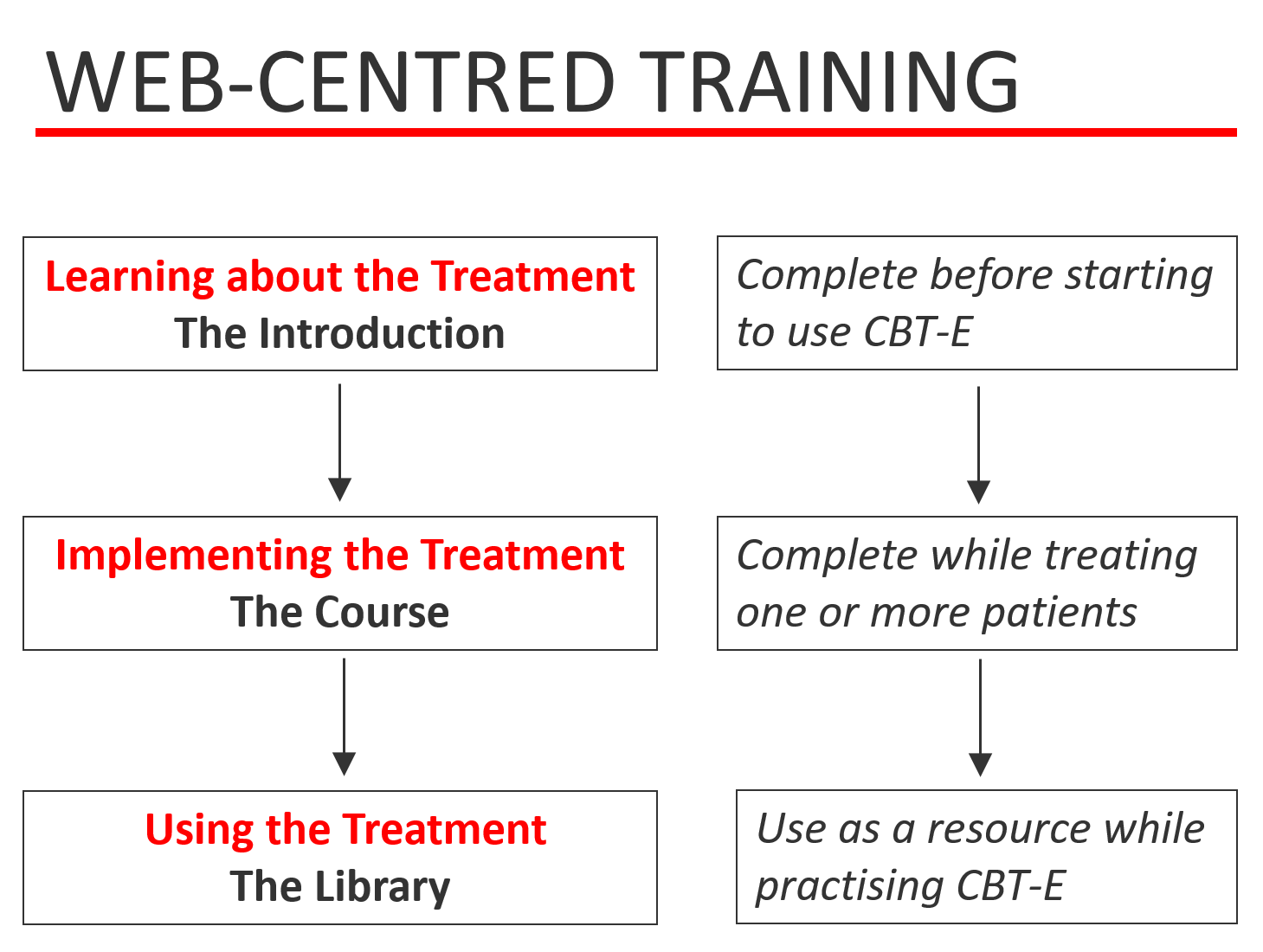

Supplement: Multimedia Appendix 3 [file jmir_v19i6e214_app3.PNG]
